# Imaging features of COVID-19-associated secondary sclerosing cholangitis on magnetic resonance cholangiopancreatography: a retrospective analysis

**DOI:** 10.1186/s13244-022-01266-9

**Published:** 2022-08-08

**Authors:** Soleen Ghafoor, Manon Germann, Christoph Jüngst, Beat Müllhaupt, Cäcilia S. Reiner, Daniel Stocker

**Affiliations:** 1 Institute of Diagnostic and Interventional Radiology, University Hospital Zurich, Rämistrasse 100, 8091 Zurich, Switzerland; 2 University of Zurich, Zurich, Switzerland; 3 Department of Gastroenterology and Hepatology, University Hospital Zurich, Rämistrasse 100, 8091 Zurich, Switzerland

**Keywords:** COVID-19, Cholangitis, Sclerosing, Cholangiopancreatography, Magnetic resonance

## Abstract

**Background:**

Despite emerging reports of secondary sclerosing cholangitis (SSC) in critically ill COVID-19 patients little is known about its imaging findings. It presents as delayed progressive cholestatic liver injury with risk of progression to cirrhosis. Diagnosis cannot be made based on clinical presentation and laboratory markers alone. Magnetic resonance imaging (MRI) and magnetic resonance cholangiopancreatography (MRCP) can aid in the diagnosis. The aim of this study was to describe MRI/MRCP imaging features of COVID-19-associated SSC.

**Results:**

Seventeen patients (mean age 60.5 years, 15 male) who underwent MRI/MRCP were included. All had been admitted to intensive care unit (ICU) (median duration of ICU stay 10 weeks, range, 2–28 weeks) and developed acute respiratory distress syndrome requiring mechanical ventilation. On imaging, all patients had intrahepatic bile duct strictures and 10 (58.8%) had associated upstream dilatation. Intrahepatic bile duct beading was seen in 14 cases (82.3%). Only one patient (5.9%) had extrahepatic bile duct stricturing. Patchy arterial phase hyperenhancement and high signal on T2- and diffusion-weighted images were seen in 7 cases (53.8%) and 9 cases (52.9%), respectively. Biliary casts were seen in 2 cases (11.8%). Periportal lymphadenopathy and vascular complications were not seen.

**Conclusion:**

On MRI/MRCP, COVID-19-associated SSC presents with multiple intrahepatic bile duct strictures with or without upstream dilatation and intrahepatic bile duct beading. Surrounding hepatic parenchymal changes including alterations in enhancement and T2 signal are common. The extrahepatic biliary tree was typically spared and periportal lymphadenopathy was missing in all patients.

**Supplementary Information:**

The online version contains supplementary material available at 10.1186/s13244-022-01266-9.

## Key points

- Secondary sclerosing cholangitis occurs in critically ill COVID-19 patients with prolonged cholestasis.
- Imaging findings on MRCP include multiple strictures of the intrahepatic bile ducts.
- Hepatic parenchymal changes and architectural distortion are commonly associated.
- Periportal lymphadenopathy and vascular complications are uncommon in COVID-19-associated SSC.

## Introduction

The severe acute respiratory syndrome coronavirus type 2 (SARS-CoV-2), the causative pathogen for the coronavirus disease 2019 (COVID-19) has caused an ongoing pandemic since its first appearance end of December 2019. Although the main symptoms of COVID-19 are of respiratory nature, ranging from mild flu-like symptoms to severe pneumonia causing acute respiratory distress syndrome (ARDS) and multi-organ failure, there are emerging reports on a wide range of extrapulmonary systemic symptoms, including hepatic and biliary complications [1–3]. Liver injury has been observed in a significant proportion of patients ranging from 16 to 45%, especially in those with a severe or critical illness and prolonged stay at the intensive care unit (ICU) [4]. Most cases of liver involvement are mainly characterized by elevated transaminases and are thought to represent a combination of direct virus-induced cholangiocyte damage, drug-induced hepatotoxicity and cytokine storm. However, a small proportion of the patients with critical illness including ARDS may develop a rapidly progressive cholestatic liver injury reminiscent of secondary sclerosing cholangitis in critically ill patients (SSC-CIP) of other causes [4–6]. The etiology of this COVID-19-associated SSC is hypothesized to be multifactorial and potential inciting factors include systemic inflammatory response syndrome (SIRS) with cytokine storm, hypoxic injury to cholangiocytes, viral-induced direct cytotoxicity, micro- and macrovascular changes leading to hypercoagulability and use of hepatotoxic medications (e.g., antibiotics, ketamine) in these critically ill patients [7]. Diagnosis is made clinically in conjunction with laboratory markers, magnetic resonance imaging (MRI) with magnetic resonance cholangiopancreatography (MRCP) and/or endoscopic retrograde cholangiopancreatography (ERCP). Liver biopsy can be performed but is not necessarily required for establishing the diagnosis in the appropriate clinical setting. If performed, biopsy will show findings including portal edema, mixed portal inflammation and pronounced bile duct damage with lobular bile infarcts and severe hepatocellular, canalicular and focally ductular cholestasis [6]. Affected patients develop progressive cholestasis and are at high risk of rapidly progressing to biliary cirrhosis requiring liver transplantation [6, 8, 9]. Several case reports and few case series have described the clinical features of this entity [6, 10–14], however, the MRI features have not yet been described in detail.

Since differential diagnosis to other causes of cholestatic liver injury can be difficult based on clinical findings and laboratory markers alone, MRI and MRCP are important diagnostic tools that provide important clues for the diagnosis of SSC in this specific setting. Hence, knowledge of the expected imaging findings will aid in making the correct diagnosis in these patients.

Therefore, the objective of this study was to describe the MR and MRCP imaging findings in patients with COVID-19-associated SSC.

## Materials and methods

Local ethics committee approval was obtained for this single-center retrospective study and need for written informed consent was waived.

### Patients

A retrospective search of the institutional radiology information system and picture archiving and communication system (PACS) between March 2020 and January 2022 was conducted to identify all consecutive critically ill patients with known COVID-19 infection (diagnosed with SARS-CoV-2 reverse transcriptase–polymerase chain reaction obtained via nasopharyngeal swabs) undergoing a liver MRI examination for unexplained abnormal liver function tests and/or clinically suspected SSC. Diagnosis of SSC was made based on clinical presentation and laboratory markers in conjunction with imaging and follow-up with or without a biopsy. More specifically, COVID-19-associated SSC was diagnosed in patients with severe SARS CoV-2 infection who presented with cholestatic liver injury and typical radiological findings in MRI and MRCP and who had no evidence of a cholestatic liver disease prior to their COVID-19-related hospital admission. Cholestasis was diagnosed through laboratory markers with or without associated signs and symptoms of cholestasis (e.g., jaundice, pruritus, fatigue). It was defined as an alkaline phosphatase (ALP) level of ≥ 1.5x the upper limit of normal (ULN) and gamma-glutamyl transferase (GGT) level of ≥ 3x ULN as suggested by the European Association for the Study of the Liver [15]. Exclusion criteria were missing MRCP and non-diagnostic image quality. The electronic medical records of the enrolled patients were reviewed. Patient demographics (age, sex), body mass index (BMI), history of underlying liver disease, presence of jaundice, symptoms of cholestasis (pruritus), laboratory data (alanine aminotransferase [ALT], aspartate aminotransferase [AST], ALP, GGT, total bilirubin) at the time of imaging and at their peak, date of initial COVID-19 diagnosis, use of ketamine, presence of ARDS diagnosis, length of ICU stay and requirement for extracorporeal membrane oxygenation (ECMO) were recorded.

### MRI protocol

MRI studies from in-house and outside hospitals were included for this study. In-house studies were performed on either a 3.0 T (Siemens Skyra, Siemens Healthineers,* n* = 10; GE Discovery MR750w, GE Healthcare, *n* = 1) or a 1.5 T scanner (Siemens Magnetom Sola, Siemens Healthineers, *n* = 1; GE Signa Artist, GE Healthcare, *n* = 1). Four studies from outside institutions were included for review, 2 were performed on a 3.0 T scanner (Philips Ingenia, Philips Healthcare, *n* = 2) and 2 on a 1.5 T scanner (Siemens Avanto fit, Siemens Healthineers, *n* = 1; Siemens Aera, Siemens Healthineers, *n* = 1). Technical details about the sequence parameters of the pulse sequences which were reviewed can be viewed in Additional file 1: Supplementary Table 1. Since MRI studies from different scanners were included, there were slight variations in scan protocols. Briefly, all included MRI studies were dedicated protocols of the liver including T2- and T1-weighted images (T2WI, T1WI) and dedicated MRCP sequences. Most studies included diffusion-weighted imaging (DWI) and the administration of either extracellular (Gadoteric acid, Dotarem^®^, Guerbet; or Gadobutrol, Gadovist^®^, Bayer Vital) or hepatospecific (Gadoxetate disodium, Primovist^®^, Bayer Vital) intravenous contrast agents. The two-point Dixon method with 3D gradient echo sequence or chemical shift imaging with in- and opposed-phase were used for assessment of steatosis in the liver. In four patients, magnetic resonance elastography (MRE) had been performed with a Resoundant driver on a 3.0 T clinical MRI system (Skyra; Siemens Healthcare) using a 2D spin-echo sequence with a fast echo-planar imaging (2D SE-EPI) readout.

### Image review

The imaging features of the first available MRI at the time of SSC diagnosis were evaluated. Images were reviewed in consensus in one session by two board-certified radiologists (C.S.R. and D.S.; reader 1 and reader 2) with 15 years and 7 years of experience in abdominal imaging. Following imaging features were assessed: changes of intrahepatic and extrahepatic bile ducts and their distribution, peribiliary changes, hepatic parenchymal changes and changes of the hepatic vessels. The imaging features which were assessed are listed in detail in Table 1. For those patients who received a concomitant MRE, the liver stiffness values were assessed by placing free-hand regions of interest on the 95% confidence stiffness maps by another radiologist (S.G., 7 years of experience in abdominal imaging). A liver stiffness of < 2.9 kPa was considered normal according to previously published values [16]. In those cases with MRE, pathology reports of liver biopsies, if available, were reviewed to correlate liver stiffness values with pathology results regarding presence of fibrosis.

**Table 1:** List of imaging features assessed on MRI and MRCP

	MRI/MRCP	Notes
Biliary tree	Extra-/intrahepatic bile duct dilatation	
	Extra-/intrahepatic bile duct strictures with or without upstream dilatation	If present, distribution was assessed as bilobar, monolobar or segmental
	Extra-/intrahepatic bile duct beading	If present, distribution was assessed as bilobar or monolobar (involving few or multiple ducts), or segmental
	Intrahepatic bile duct saccular dilatation	If present, distribution was assessed as bilobar or monolobar (involving few or multiple ducts), or segmental
	Vanishing ducts	If present, distribution was assessed as bilobar, monolobar or segmental
	Extra-/intrahepatic intrabiliary casts	If present, distribution was assessed as bilobar, monolobar or segmental
	Extra-/intrahepatic periportal and/or peribiliary signal changes	Hyperintense signal changes were assessed on T2-weighted sequences and diffusion-weighted sequences. If present, distribution was assessed as bilobar, monolobar or segmental
	Extra-/intrahepatic periportal and/or peribiliary enhancement	Enhancement was assessed on multiphasic post-contrast T1-weighted images. If present, distribution was assessed as bilobar, monolobar or segmental
	Presence of gall bladder sludge and/or stones	
Hepatic parenchyma	Hepatomegaly	Feature was assessed qualitatively. Measurements of the maximum craniocaudal length of the liver in the coronal plane were performed for orientation (cutoff: 16 cm). Other signs included extension of the right lobe beyond the lower pole of the right kidney and rounded contour of the inferior hepatic border
	Distortion of the liver morphology	Features were assessed qualitatively and included structural changes of the liver contour such as a rounded shape of the liver, caudate lobe hypertrophy, lobar hypertrophy or atrophy A cirrhotic morphology was defined as a combination of signs including widening of the porta hepatis, enlargement of the interlobar fissure, expansion of pericholecystic space, segmental atrophy (segment 4), compensatory hypertrophy (segments 2 and 3, caudate lobe), a nodular liver contour and heterogeneity of the liver
	Hepatic parenchymal signal changes	Signal changes were assessed on T2-weighted sequences and diffusion-weighted sequences
	Steatosis	Feature was assessed using the 2-point Dixon method or chemical shift imaging with in- and opposed-phase and was defined as a calculated liver fat fraction exceeding 5%. If present, distribution was described as either focal or diffuse
Vascular	Thrombosis or occlusion	Changes of the hepatic arteries, the portal vein and hepatic veins were assessed
	Caliber irregularities and strictures	
Other	Ascites	If present, it was quantified subjectively as small volume, moderate volume or large volume
	Portal and/or portocaval lymphadenopathy	Defined as enlarged lymph nodes ≥ 1 cm short axis

### Statistical analysis

Descriptive statistics for the clinical and imaging features were performed. Categorical variables were described as frequencies and percentages. Continuous variables were described as means and standard deviations or medians and range.

## Results

### Patients and clinical characteristics

Our search identified 18 patients with clinical suspicion of COVID-19-associated SSC who had undergone a liver MRI between March 2020 and January 2022 at our institution or at outside institutions with the imaging studies imported into our PACS. One patient was excluded due to lack of MRCP sequences. The remaining 17 cases (15 male, 2 female) were included for further analysis.

Mean age was 60.5 years (± 9.9 years). Median BMI was 26.7 kg/m^2^ (range, 19.8–43.9 kg/m^2^). Three patients (17.6%) had a history of prior liver disease: One patient had a history of isolated asymptomatic GGT elevation; one patient had a history of viral hepatitis A and hepatitis B infection, and one patient had non-alcoholic steatohepatitis. All patients had been admitted to ICU with a median length of ICU stay of 10 weeks (range, 2–28 weeks).

All patients had developed ARDS requiring mechanical ventilation and 6 patients (35.3%) had required ECMO support. Four patients (23.5%) underwent liver transplantation due to SSC, and one additional patient was under pre-transplant evaluation at the time of data accrual.

The detailed clinical variables including the pertinent laboratory markers are listed in Table 2.

**Table 2:** Demographics and clinical variables

	Total *n* = 17
Age [years]	60.5 (± 9.9)
Gender [*n*]	
Male	15 (88.2%)
Female	2 (11.8%)
BMI [kg/m^2^]	26.7 (19.8–43.9)
– Overweight (BMI 25–30 kg/m^2^)	9 (52.9%)
– Obese (BMI ≥ 30 kg/m^2^)	3 (17.6%)
Jaundice [*n*]	3 (17.6%)
Pruritus [*n*]	5 (29.4%)
Length of ICU stay [weeks]	10 (2–28)
ARDS [*n*]	17 (100%)
ECMO support [*n*]	6 (35.3%)
Ketamine [*n*]*	7 (41.2%)
Preexisting liver disease [*n*]	3 (17.6%)
– NASH	1
– Isolated GGT elevation	1
– History of hepatitis A and hepatitis B infection	1
Abnormal liver function tests [*n*]	17 (100%)
Laboratory markers (at time of imaging)	
– Aspartate aminotransferase (AST)	143.7 U/l (± 117.0) [x2.9 ULN (± 2.3)]
– Alanine aminotransferase (ALT)	158.1 U/l (± 118.5) [x3.2 ULN (± 2.4)]
– Gamma glutamyltransferase (GGT)	1365.1 U/l (± 1579.8) [x10.3 ULN (± 8.6)]
– Alkaline phosphatase (ALP)	620.2 U/l (± 516.3) [x4.8 ULN (± 4.0)]
– Bilirubin (total)	97.4 µmol/l (± 134.5) [x4.6 ULN (± 6.4)]
Laboratory markers (peak)	
– Aspartate aminotransferase (AST)	334.1 U/l (± 419.9) [x6.7 ULN (± 8.4)]
– Alanine aminotransferase (ALT)	367.1 U/l (± 412.4) [x7.3 ULN (± 8.2)]
– Gamma glutamyltransferase (GGT)	1908.8 U/l (± 1327.6) [x31.8 ULN (± 22.1)]
– Alkaline phosphatase (ALP)	1181.5 U/l (± 1044.4) [x9.1 ULN (± 8.0)]
– Bilirubin (total)	140.5 U/l (± 200.2) [x6.7 ULN (± 9.5)]

Continuous variables are presented as either mean (± standard deviation) or median (range). Categorical variables are presented as counts (percentage)

*ICU* intensive care unit, *ARDS* acute respiratory distress syndrome, *ECMO* extracorporeal membrane oxygenation, *NASH* non-alcoholic steatohepatitis, *GGT* gamma glutamyltransferase, *ULN* upper limits of normal

*This information could not be obtained in 7 patients, as they had been treated at outside hospitals and detailed ICU reports were not available for review

In 13 of the 17 cases the MRI exam was performed with administration of an intravenous contrast agent, 4 cases were non-contrast studies. Seven patients received a hepatospecific contrast agent (Gadoxetate disodium, Primovist^®^, Bayer HealthCare) and 6 patients received an extracellular contrast agent (*n* = 5, gadoteric acid, Dotarem^®^, Guerbet; *n* = 1, gadobutrol, Gadovist^®^, Bayer HealthCare).

### Imaging findings

#### Biliary tree imaging findings

##### Intrahepatic biliary changes

**Fig. 1:**
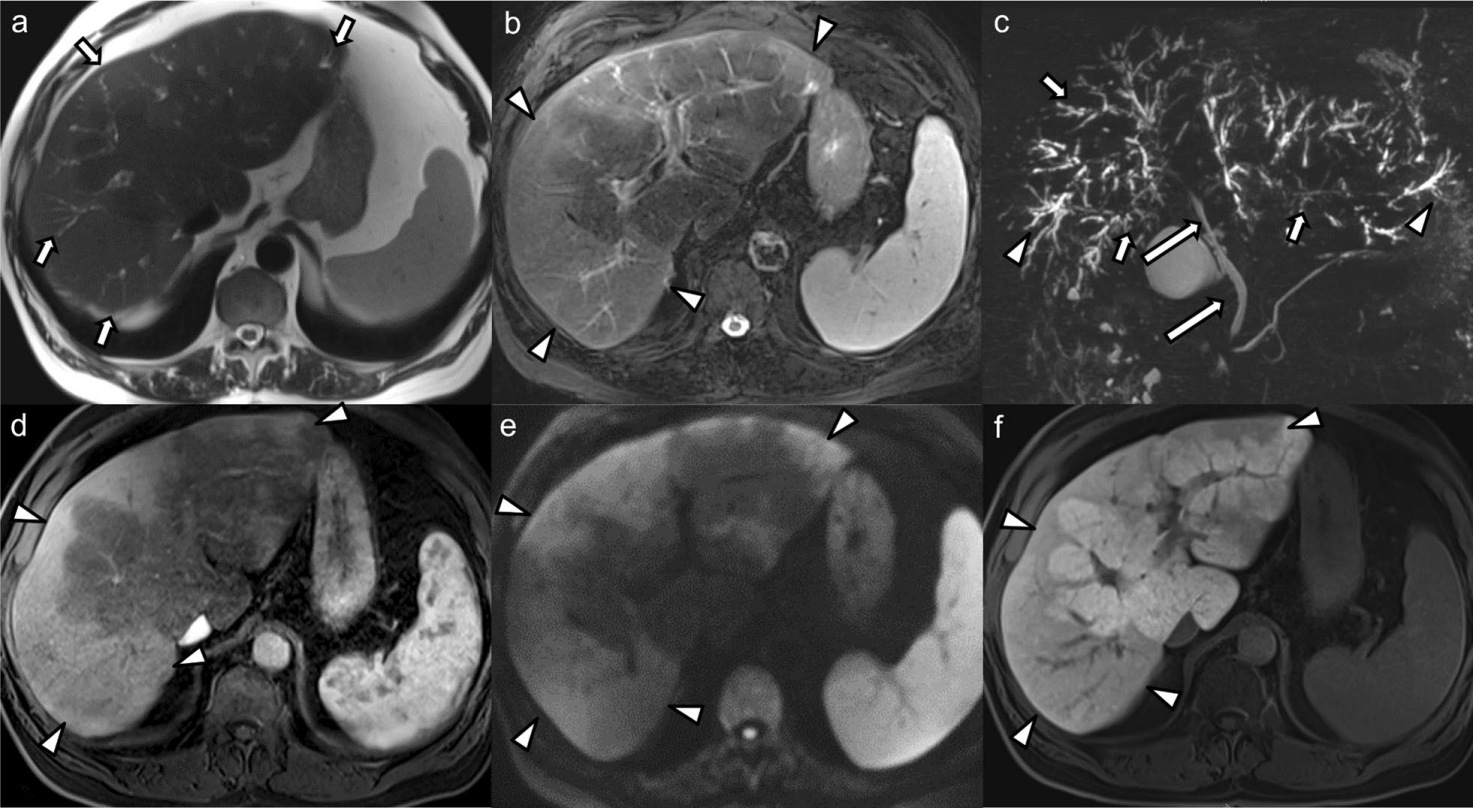
70-year-old male patient who developed generalized pruritus and laboratory signs of hepatopathy a few months after recovering from a severe COVID-19 infection. MRI and MRCP of the liver with gadoxetate disodium was performed: **a** Axial T2-weighted image shows multifocal peripheral areas of mild biliary ductal dilatation and faint hyperintense parenchymal signal changes (arrows). **b** Peripheral parenchymal hyperintense signal changes are better seen on the fat-suppressed T2-weighted image (arrowheads). **c** Maximum intensity projection MRCP shows multiple strictures of the intrahepatic bile ducts (arrows) with upstream dilatation (arrowheads). The extrahepatic bile duct is spared (long arrows). **d** Post-contrast image in the arterial phase shows hyperenhancement in the peripheral parenchymal areas with hyperintense signal on high b-value diffusion-weighted images (**e**) and hypointensity in the hepatobiliary contrast phase (**f**) (arrowheads in **d**–**f**). Biopsy showed findings consistent with chronic cholestatic hepatopathy, bile duct damage and Stage F3 fibrosis

**Fig. 2:**
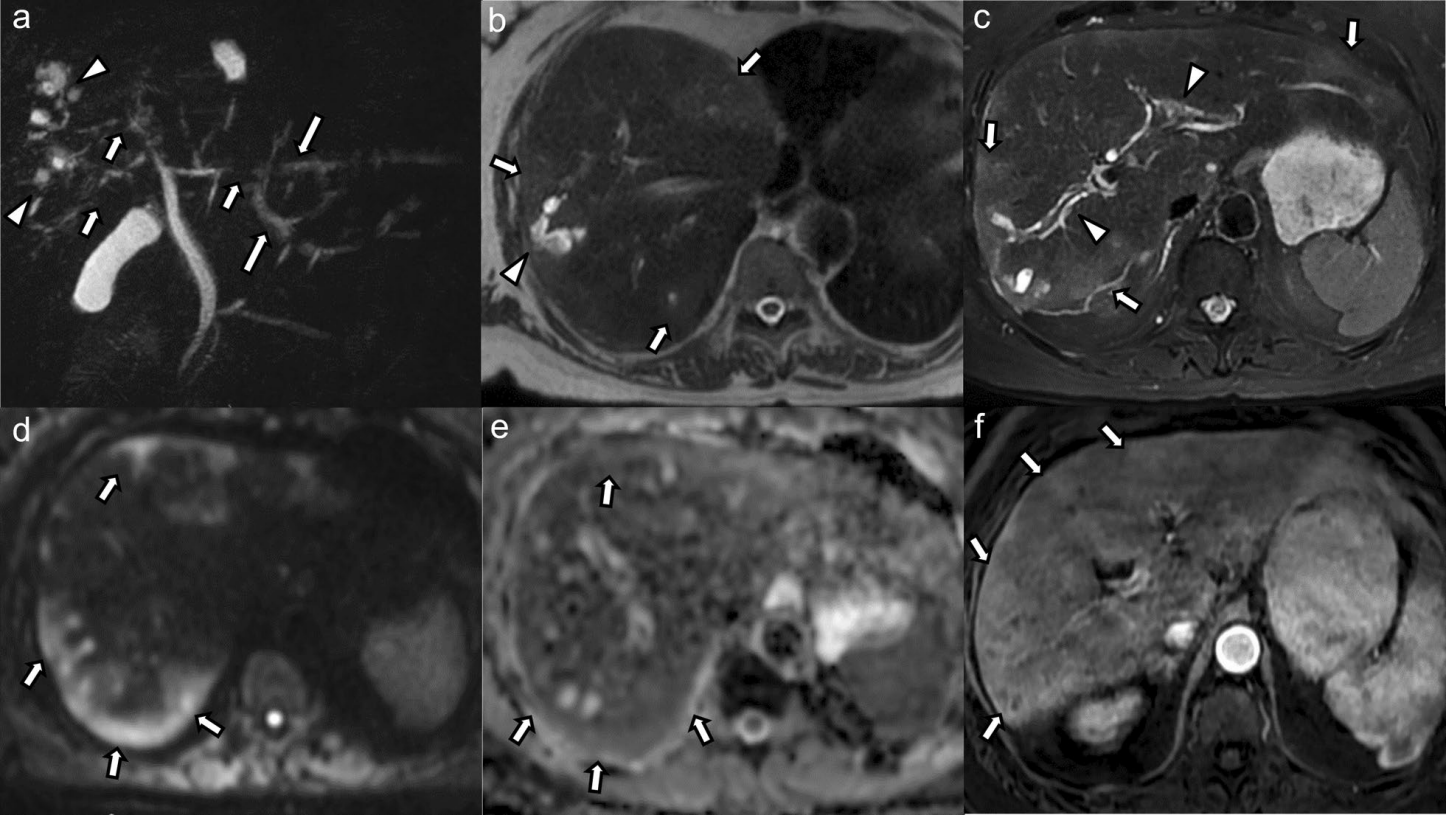
69-year-old male patient with COVID-19-associated SSC complicated by acute bouts of cholangitis with development of multiple biliary abscesses requiring prolonged antibiotic and interventional therapy. **a** Maximum intensity projection MRCP image shows multifocal areas of bile duct stricturing (arrows) in both liver lobes with intermittent mild upstream dilatation (long arrows). In the right liver lobe, there are clustered cystic changes representing saccular dilatation of bile ducts (arrowheads). **b** Axial non-fat-suppressed and (**c**) fat-suppressed T2-weighted images show multiple patchy hyperintense parenchymal changes in the periphery and subcapsular liver (arrows in b and c). Saccular dilatation of peripheral bile ducts (arrowhead in **b**) with intraluminal debris. There are hyperintense peribiliary signal changes (arrowheads in **c**). **d** High *b*-value diffusion-weighted images and (**e**) corresponding ADC map again show these peripheral and subcapsular signal changes with mild diffusion restriction (arrows). **f** On post-contrast arterial phase images these parenchymal changes are hyperenhancing (arrows)

All patients had intrahepatic bile duct strictures (bilobar *n* = 14, monolobar *n* = 2, segmental *n* = 1), of which 10 patients (58.8%) had upstream dilatation (bilobar *n* = 2, monolobar *n* = 6, segmental *n* = 2) (Fig. 1). Seven patients (41.2%) had intrahepatic bile duct dilatation unrelated to strictures (bilobar *n* = 2, monolobar *n* = 4, segmental *n* = 1). Intrahepatic bile duct beading was seen in 14 cases (82.3%), of those 4 (28.6%) were bilobar involving a few ducts, 6 (42.8%) bilobar involving multiple ducts, 2 (14.3%) monolobar involving a few ducts, and 2 (14.3%) monolobar involving multiple ducts. Saccular dilatation of the intrahepatic bile ducts was seen in 5 cases (29.4%), of which 4 cases were bilobar and 1 case was monolobar (Fig. 2). Vanishing ducts were seen in 8 cases (47.1%), the majority (87.5%) of which were bilobar.

##### Extrahepatic biliary changes

None of the patients had extrahepatic bile duct dilatation. Only one patient (5.9%) had extrahepatic bile duct stricturing without upstream dilatation. There was no case of extrahepatic bile duct beading or saccular dilatation.

##### Peribiliary changes

On T2WI and DWI, hyperintense peribiliary signal changes of the extrahepatic bile duct were seen in only one case (5.9%) whereas these changes were present around the intrahepatic bile ducts in 12 cases (70.6%), of which the majority (91.7%) involved both hepatic lobes.

In those cases where MRI was performed with intravenous contrast agents (*n* = 13), peribiliary enhancement involving the extrahepatic bile ducts was seen in only one case (7.7%) and in 3 cases (23.1%) involving the intrahepatic bile ducts.

##### Other biliary findings

Biliary casts were seen in two cases (11.8%), one case involved the intra- and extrahepatic bile ducts and one case the extrahepatic bile duct only. In one other case (5.9%) sludge was noted in the extrahepatic biliary tree. Gallbladder sludge was seen in 2 cases (11.8%) and three cases (17.6%) had gallbladder stones. Biliary abscesses were seen in one case (5.9%).

#### Hepatic parenchymal imaging findings

Hepatomegaly was seen in 4 cases (23.5%). Ten patients (58.8%) presented with structural changes of the liver. Following structural changes were seen: rounded liver contours (*n* = 5), caudate lobe hypertrophy (*n* = 5), hypertrophy of the left liver lobe (*n* = 2), segment 4 atrophy (*n* = 1), atrophy of the peripheral liver parenchyma with compensatory central hypertrophy (*n* = 1), and cirrhotic liver morphology (*n* = 1).

**Fig. 3:**
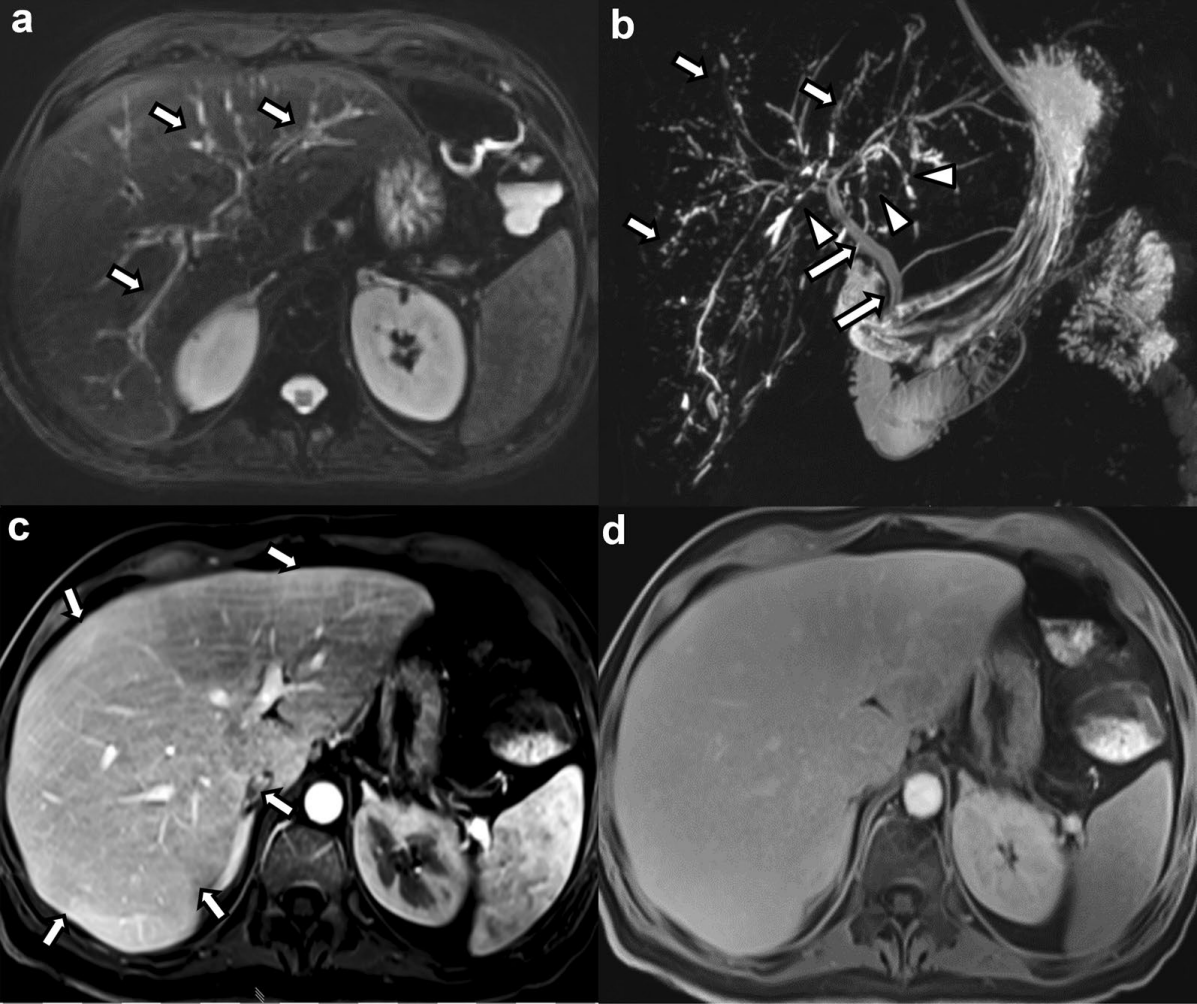
40-year-old male patient with COVID-19-associated SSC after severe COVID-19 infection with ARDS and ECMO requirement. **a** Axial fat-suppressed T2-weighted image shows mildly accentuated intrahepatic bile ducts with hyperintense peribiliary signal changes (arrows). **b** Maximum intensity projection MRCP image shows multifocal beading of the intrahepatic bile ducts (arrows) and multiple short-segment strictures (arrowheads). The extrahepatic biliary tree is spared (long arrows). **c** Post-contrast subtraction image in the arterial phase shows subtle inhomogeneous parenchymal enhancement with areas of hyperenhancement (arrows). **d** Hepatobiliary phase image acquired 20 min. after intravenous administration of gadoxetate disodium shows decreased hepatobiliary uptake with decreased liver-to-vessel-contrast. The patient underwent orthotopic liver transplantation and explant pathology revealed findings consistent with a severe ischemic cholangiopathy and grade F2 fibrosis

Irregular patchy high T2 signal and DWI changes were present in 9 cases (52.9%). In those cases where MRI was performed with intravenous contrast agents, patchy arterial phase hyperenhancement (APHE), either heterogeneous or subcapsular, was seen in 7 cases (53.8%). Areas of reduced signal intensity in the hepatobiliary phase (HBP) were seen in 6 of the 7 patients (85.7%) who underwent imaging with a hepatospecific contrast agent, of which 4 cases showed patchy areas of decreased signal and 2 cases showed diffusely and globally decreased uptake of the hepatobiliary contrast agent (Fig. 3). Three cases (17.6%) did not present with any hepatic parenchymal changes.

Hepatic steatosis was seen in 2 cases, of which one presented with diffuse steatosis and one case showed central and periportal steatosis.

#### Vascular findings

No cases of vascular thrombosis or abnormalities of the hepatic arteries, portal vein or hepatic veins were seen.

#### Other findings

**Fig. 4:**
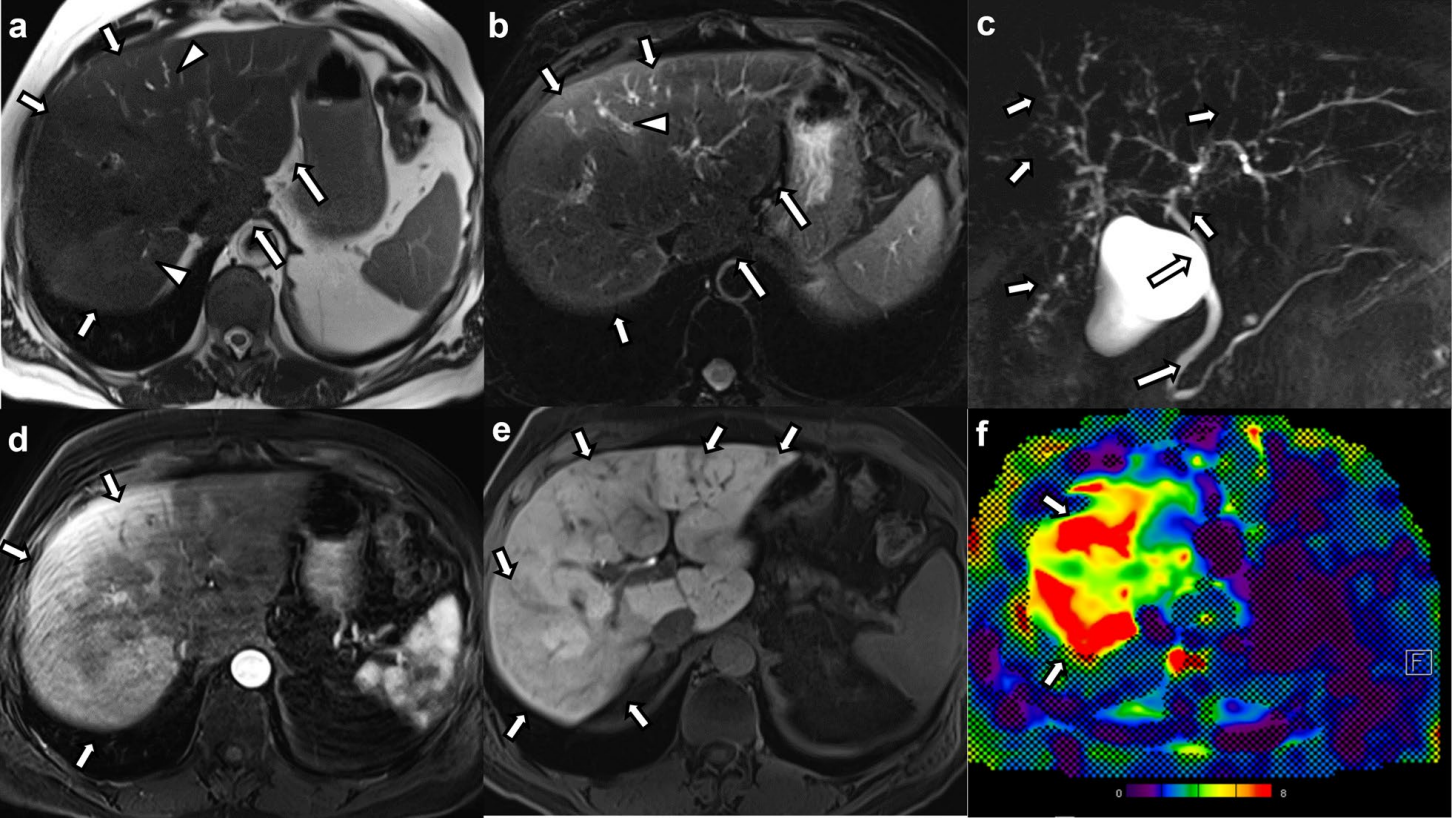
61-year-old male patient with COVID-19-associated SSC after severe COVID-19 infection and prolonged ICU stay. **a** Axial non-fat-suppressed and (**b**) fat-suppressed T2-weighted images show mild hyperintense signal changes predominantly in the right hepatic lobe (arrows in a and b) with subtle bile duct dilatation (arrowheads in **a** and **b**). The caudate lobe is enlarged, and the hepatic contours are rounded (long arrows in **a** and **b**). **c** Maximum intensity projection MRCP image shows irregularities of the intrahepatic bile ducts with beading and multifocal structuring (arrows). The extrahepatic biliary tree is spared (long arrows). **d** Post-contrast images in the arterial phase show diffuse areas of patchy hyperenhancement especially in peripheral areas of the right hepatic lobe (arrows). **d** The post-contrast images in the hepatobiliary phase show patchy hypointense changes representing areas of decreased of hepatobiliary contrast agent uptake (arrows). **f** Color stiffness map from MR elastography shows increased stiffness of the liver parenchyma (arrows) as represented by the yellow to red color coding (mean liver stiffness was 6.4 kPa)

Small volume ascites was seen in two cases (11.8%). Periportal and/or portocaval lymphadenopathy was seen in none of the cases. Four patients had MRE, and all patients showed elevated liver stiffness with values of 3.2 kPa, 4.2 kPa, 6.4 kPa, and 11.4 kPa (Fig. 4).

Three of those patients receiving MRE also underwent a liver biopsy. The parenchymal signal changes and structural changes of the liver seen on imaging in those 4 MRE cases and degree of fibrosis from pathology results are depicted in Table 3.

**Table 3:** Hepatic parenchymal changes and pathology results in the patients with MR elastography

	Hepatic parenchymal changes on imaging	Pathology findings (fibrosis grade)
Case 1 (3.2 kPa)	Patchy arterial phase hyperenhancement Hyperintense signal changes on DWI Diffusely reduced signal in the hepatobiliary phase	Mild portal and periportal fibrosis without bridging septae (F1)
Case 2 (4.2 kPa)	Patchy reduced signal in the hepatobiliary phase	Portal fibrosis, mild pericentral and pericellular fibrosis without bridging septae (F1)
Case 3 (6.4 kPa)	Patchy arterial phase hyperenhancement Patchy peripheral and subcapsular hyperintense signal changes on T2-weighted images Patchy reduced signal in the hepatobiliary phase Rounded liver contours Caudate lobe hypertrophy	N/A
Case 4 (11.4 kPa)	Patchy arterial phase hyperenhancement Caudate lobe hypertrophy	Portal and pericellular fibrosis without bridging septae (F1)

The depicted kPa values correspond to the liver stiffness values assessed with MR elastography

Cases 1, 2 and 3 received MRI with the hepatospecific contrast agent gadoxetate disodium, Case 4 received the extracellular contrast agent gadoteric acid

## Discussion

In this retrospective descriptive study, we evaluated the MR and MRCP imaging findings in 17 patients with COVID-19-associated SSC. This is to date the largest case series on the imaging findings in this emerging entity. We found that all patients present with intrahepatic bile duct strictures on MRCP with or without associated biliary ductal dilatation. The majority of these cases presented with a beaded appearance of the bile ducts and nearly half of the cases with a “pruned tree” appearance due to vanishing peripheral ducts. These changes involved more than one hepatic segment (commonly bilobar) and were commonly associated with peribiliary signal changes and signal changes in the hepatic parenchyma.

COVID-19 infection can result in a number of extrapulmonary manifestations including hepatobiliary injury [17]. The widening recognition of abdominal manifestations of COVID-19 has led to a few studies and case reports on the imaging manifestations in the abdomen [18–20], but most of these data focused on CT and included mainly luminal and vascular gastrointestinal abnormalities. While clinical reports have increasingly identified hepatobiliary manifestations of COVID-19, their radiological manifestations are largely unknown and underreported. The current data on COVID-19-associated hepatobiliary manifestations mainly stems from case reports from the gastroenterological literature and detailed description of their imaging manifestations is lacking [6, 10, 21, 22]. Specifically, development of SSC in COVID-19 patients has been described only recently and is akin to SSC-CIP of other causes, presenting as a delayed extrapulmonary COVID-19 manifestation characterized by progressive cholestatic liver injury with high risk of rapid progression to biliary cirrhosis [6].

The underlying pathophysiology in patients with SSC-CIP is not fully understood but is likely multifactorial (ischemic cholangiopathy and so called “toxic bile”) leading to progressive cholestasis, cholangiocyte necrosis, and progression to secondary biliary cirrhosis [7, 23]. Common-risk factors of SSC-CIP are encountered in critically ill patients requiring ICU care (systemic hypotension, vasopressor support, mechanical ventilation with high positive end-expiratory pressures, prone positioning, and drug-induced cytotoxic effects) [7, 11, 23]. The patients in our cohort displayed several risk factors for the development of SSC-CIP as they all developed a critical illness requiring ICU care, were all mechanically ventilated and received multiple medications. Notably, at least more than a third of the patients also received ketamine, a sedative agent which has been associated with the development of secondary sclerosing cholangitis in the setting of recreational and therapeutic use [24–26].

A recent retrospective single-center study analyzed all consecutive COVID-19 patients admitted to the ICU during a two-month period using a historic comparative cohort of critically ill ICU patients with influenza A and found a relatively high prevalence of 12% for SSC-CIP in the COVID-19 cohort compared to no cases in the influenza A-cohort [6]. Furthermore, all the patients who developed SSC in this cohort had received ketamine. In addition to drug-induced toxicity, these findings, although preliminary, could also indicate potential specific COVID-19-associated factors besides the known SSC-CIP-risk factors that promote the development of SSC in these patients. Hence, post–COVID-19 cholangiopathy could represent a confluence of SSC-CIP and direct hepatobiliary injury from COVID-19 [9].

In our study we observed signal changes including peribiliary edema, parenchymal high signal intensity changes on T2WI and DWI, inhomogeneous APHE and decreased enhancement in the hepatobiliary phase. Like primary sclerosing cholangitis (PSC), these liver parenchymal signal changes are thought to be secondary to reduced bile stream, focal areas of superimposed cholangitis and developing fibrosis. The imaging findings overlap with PSC and SSC of other causes [27, 28], however, we believe this is unlikely to pose a diagnostic dilemma given the specific clinical scenario and patient history in cases of COVID-19-associated SSC. Of note, while periportal lymphadenopathy is a characteristic feature in PSC, it was not seen in our cases of COVID-19-associated SSC [29, 30]. Ultimately, the imaging findings in our cohort of COVID-19-associated SSC do not differ from previously described imaging findings of SSC from other causes, hence, the clinical context is essential in the differential diagnosis [31–33].

Involvement of the extrahepatic biliary tree was only present in one case in our study. This pattern of preferential involvement of the intrahepatic biliary tree and sparing of the extrahepatic bile ducts is distinctive from PSC but similar to sclerosing cholangitis due to ischemic cholangiopathy or SSC-CIP. While important contributing factors in PSC and IgG4-related sclerosing cholangitis are immune-mediated and related to a sustained inflammation, the pathogenesis of ischemic cholangiopathy and SSC-CIP is linked to bile duct ischemia [23, 34, 35]. The singular arterial supply of the intrahepatic bile ducts via the hepatic artery renders them especially susceptible to hypoxia and ischemia, whereas the extrahepatic biliary tree receives a dual arterial supply via the hepatic artery and gastroduodenal artery and is likely less susceptible in a native liver [36].

Interestingly, intrabiliary casts were seen in only two cases in our cohort. Formation of biliary casts occurs mainly in patients post orthotopic liver transplant (OLT) and is associated with ischemic injury to the biliary epithelium. It is estimated to occur in 3–18% in patients with OLT [37]. In native livers, biliary cast formation is rare, but has been described as a hallmark of SSC-CIP [5]. Several reports have described the presence of intrabiliary cast in cases COVID-19-associated SSC [7, 12, 21, 22]. However, in some of these reports the casts were detected on ERCP. Our findings showed only a very low prevalence of intrabiliary casts on imaging. We hypothesize that the low prevalence of intrabiliary casts on MRCP in our study could be related to the fact that MRCP could potentially be missing the presence of smaller casts, especially in cases of non-dilated ducts, involvement of the smaller intrahepatic bile ducts and lack of associated T1-hyperintensity [38]. Although the presence of biliary casts has been described to be characteristic for non-COVID-19-related SSC-CIP (occurring in up to 87%), our findings indicate that lack thereof does not exclude it [5].

COVID-19 is associated with vascular complications including increased risk of micro- and macrovascular thromboembolism [39]. Several alterations in the coagulation pathway promoting a hypercoagulable state have been linked to COVID-19-associated coagulopathy [40]. In the abdomen, findings of bowel ischemia due to mesenteric and portal vein thrombosis, arterial mesenteric thrombosis and arterial dissections of the splanchnic vessels have been described [41–44]. Few cases of hepatic macrovascular complications including hepatic artery thrombosis and portal vein thrombosis have also been reported [45, 46]. However, hepatic macrovascular involvement seems a rare event and none of the patients in our cohort presented with macrovascular changes on imaging. Nevertheless, involvement of the microvascular bed (which cannot be confidently assessed on imaging) is very plausible given thepathogenetic pathways linked to ischemic cholangiopathy and bile duct ischemia in SSC-CIP.

Four patients underwent MRE, and all showed elevated liver stiffness ranging from mildly to markedly elevated values. In addition to tissue composition, vascular-related factors and interstitial pressure can affect liver parenchymal stiffness [47]. Other pathologic processes that can also cause increased liver stiffness include inflammation, biliary obstruction and cholestasis, passive congestion, and increased portal venous pressure [47, 48]. Although the available pathology results from biopsies indicated the presence of mild fibrosis, we are ultimately not able to clearly define the causative factors for the variably elevated liver stiffness values on MRE in our four cases. Nevertheless, SSC-CIP has been reported to be associated with a high risk of rapid progression to liver cirrhosis requiring OLT with shorter median liver transplantation-free survival rates than PSC [5, 49].

At the time of data analysis, four patients had undergone liver transplantation and one patient was undergoing pre-transplant assessment. On imaging, nearly 60% of patients presented with structural changes of the liver indicating architectural distortion and remodeling because of developing fibrosis. Concomitantly, the majority (> 80%) presented with hepatic parenchymal changes including high signal intensity changes on T2WI and DWI, areas of heterogeneous APHE, and patchy or diffusely reduced signal intensity in HBP.

In our cohort we could observe a predominance of male patients. This appears to be in line with other published reports on COVID-19-associated SSC [6, 10, 11, 13, 50]. Furthermore, male gender is a known risk factor for the development of severe COVID-19 [51].

Our study has some limitations. First, the retrospective design of this single-center study entails biases, most notably a selection bias. However, we used data from consecutive patients to reduce this bias. Second, the relatively small case number did not allow for advanced statistical analysis and potential correlation of imaging findings with outcomes. However, to the best of our knowledge, this is to date the largest case series on the imaging findings in patients with COVID-19-associated SSC. Third, MRI protocols were not homogeneous and MRI studies with extracellular and hepatospecific contrast agents were assessed. However, since the focus of this study was on descriptive features and not quantitative parameters, we believe this should not be a major limitation with regard to the study objective. Lastly, image analysis was done based on a consensus reading, this does not permit analysis of inter-reader agreement and reproducibility.

## Conclusion

COVID-19-associated SSC presents with multiple intrahepatic bile duct strictures and surrounding hepatic parenchymal changes including alterations in enhancement, T2 signal and distortion of liver architecture. The extrahepatic biliary tree is spared in most cases and periportal lymphadenopathy is lacking. Macrovascular complications are not seen. Knowledge of the findings of COVID-19-associated SSC on MRI/MRCP can help radiologists identify this novel entity and guide diagnosis.

## Supplementary Information

**Additional file 1. Supplementary Table 1**. Sequence parameters of analyzed MRI studies.

## Data Availability

The datasets generated and/or analyzed during the current study are not publicly available due to privacy and data confidentiality reasons but are available from the corresponding author on reasonable request.

## Abbreviations

ARDS: Acute respiratory distress syndrome
ALT: Alanine aminotransferase
ALP: Alkaline phosphatase
ACE2: Angiotensin-converting enzyme 2
APHE: Arterial phase hyperenhancement
AST: Aspartate aminotransferase
BMI: Body mass index
COVID-19: Coronavirus disease 2019
DWI: Diffusion-weighted imaging
ERCP: Endoscopic retrograde cholangiopancreatography
ECMO: Extracorporeal membrane oxygenation
GGT: Gamma-glutamyl transferase
HBP: Hepatobiliary phase
ICU: Intensive care unit
MRCP: Magnetic resonance cholangiopancreatography
MRE: Magnetic resonance elastography
MRI: Magnetic resonance imaging
OLT: Orthotopic liver transplant
PACS: Picture archiving and communication system
PSC: Primary sclerosing cholangitis
RIS: Radiology information system
SSC: Secondary sclerosing cholangitis
SSC-CIP: Secondary sclerosing cholangitis in critically ill patients
SARS-CoV-2: Severe acute respiratory syndrome coronavirus type 2
SIRS: Systemic inflammatory response syndrome
T2WI, T1WI: T2- and T1-weighted images
ULN: Upper limit of normal
