# Supplementary material for: Imaging features of COVID-19-associated secondary sclerosing cholangitis on magnetic resonance cholangiopancreatography: a retrospective analysis
Source: Insights Imaging. 2022 Aug 8;13:128. doi: 10.1186/s13244-022-01266-9 (PMC9358102; doi:10.1186/s13244-022-01266-9)
Supplement: Supplementary file 1 — Additional file 1. Supplementary Table 1. Sequence parameters of analyzed MRI studies. [file 13244_2022_1266_MOESM1_ESM.pdf]

ELECTRONIC SUPPLEMENTARY MATERIAL

Imaging features of COVID-19 associated secondary sclerosing cholangitis on magnetic resonance cholangiopancreatography – a retrospective analysis

Supplementary Table 1. Sequence parameters of analyzed MRI studies

Sequence	TR (ms)	TE (ms)	Slice thickness (mm)	Slice gap (mm)	Image slices (n)	Flip angle (°)	Matrix size	FOV (mm)	Signal averages (n)	Echo train length	Image plane	b-values (low/ high)
3D MRCP	1024-6015	600-997	1-2.2	0-1.1	64-124	80-140	320x320-768x768	260x260-380x380	1-2	100-290	coronal	
T2-weighted image	406-3276	80-110	4-7	5.5-8	30-47	90-180	256x256-560x560	310x380-420x420	0.65-1	1-134	axial or coronal	
Fat-suppressed T2-weighted image	2177-9230	78-113	5	5-6.5	27-46	90-160	256x256-512x512	380x380-420x420	1-2	21-50	axial	
T1-weighted image	2.77-6.79	1.26-4.75	3-5	1.5-2.5	72-140	9-20	280-320-512x512	310x380-450x450	0.7-1	1-78	axial	
Diffusion-weighted image	1687-6900	52-81	5-6	5.5-7.2	36-78	90	240x240-336x336	285x380-450x450	1-8	1-67	axial	0-50/800

Abbreviations:
MRCP = Magnetic resonance cholangiopancreatography
TR = Repetition time
TE = Echo time
FOV = Field of view

Insights Imaging (2022) Ghafoor S, Germann M, Jüngst C, Müllhaupt B, Reiner CS, Stocker D
